# Variation in nomenclature of somatic variants for selection of oncological therapies: Can we reach a consensus soon?

**DOI:** 10.1002/humu.23926

**Published:** 2019-10-14

**Authors:** Cleo Keppens, Véronique Tack, Kelly Dufraing, Etienne Rouleau, Marjolijn J. L. Ligtenberg, Ed Schuuring, Elisabeth M. C. Dequeker

**Affiliations:** ^1^ Department of Public Health and Primary Care, Biomedical Quality Assurance Research Unit University of Leuven Leuven Belgium; ^2^ Gustave Roussy Service de Génétique/Pathologie Moléculaire Villejuif Cedex France; ^3^ Department of Pathology and Department of Human Genetics Radboud University Medical Center Nijmegen The Netherlands; ^4^ University Medical Center Groningen, Department of Pathology University of Groningen Groningen The Netherlands

**Keywords:** biomarker variant reporting, colorectal cancer, external quality assessment, HGVS recommendations, lung cancer, nomenclature, proficiency testing, round robin

## Abstract

A standardized nomenclature for reporting oncology biomarker variants is key to avoid misinterpretation of results and unambiguous registration in clinical databases. External quality assessment (EQA) schemes have revealed a need for more consistent nomenclature use in clinical genetics. We evaluated the propensity of EQA for improvement of compliance with Human Genome Variation Society (HGVS) recommendations for reporting of predictive somatic variants in lung and colorectal cancer. Variant entries between 2012 and 2018 were collected from written reports and electronic results sheets. In total, 4,053 variants were assessed, of which 12.1% complied with HGVS recommendations. Compliance improved over time from 2.1% (2012) to 22.3% (2018), especially when laboratories participated in multiple EQA schemes. Compliance was better for next‐generation sequencing (20.9%) compared with targeted techniques (9.8%). In the 1792 reports, HGVS recommendations for reference sequences were met for 31.9% of reports, for 36.0% of noncommercial, and 26.5% of commercial test methods. Compliance improved from 16.7% (2012) to 33.1% (2018), and after repeated EQA participation. EQA participation improves compliance with HGVS recommendations. The residual percentage of errors in the most recent schemes suggests that laboratories, companies, and EQA providers need to collaborate for additional improvement of harmonization in clinical test reporting.

## Graphical abstract

Participation to external quality assessment (EQA) improved compliance with Human Genome Variation Society (HGVS) recommendations. Compliance of variant descriptions was better for next‐generation sequencing (20.9%) compared with targeted techniques (9.8%). A correct reference sequence was included for 36.0% of noncommercial versus 26.5% of commercial test methods. The residual percentage of noncompliance with HGVS suggests that laboratories, companies, and EQA providers need to collaborate for additional harmonization in clinical test reporting.

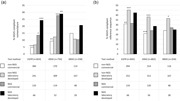

## 1 INTRODUCTION

Reporting of oncology biomarker tests in a standardized format is key to avoid misinterpretation of analysis results in clinical diagnostic reports for diagnosis and treatment decisions. Harmonized nomenclature is also necessary for unambiguous registration of variants in clinical databases (Yen et al., 2017). Altering variant descriptions could influence patient selection for treatment or clinical trials, ultimately impacting on their outcomes (Callenberg et al., 2018).

In 1998, the Human Genome Variation Society (HGVS) proposed a set of nomenclature recommendations (Antonarakis, 1998). The first version of the guidelines was published in 2000 (J. T. den Dunnen & Antonarakis, 2000). With the widespread implementation of high‐throughput sequencing and increasing complexity of detected variants, the recommendations were updated in 2016. This resulted in the current version, 19.01, publicly available from http://varnomen.hgvs.org (Johan T. Den Dunnen et al., 2016). Examples of 2016 updates include (a) the addition of brackets when amino acid changes are predicted without experimental evidence, (b) the use of the term “variant” instead of “mutation,” (c) the use of “Ter” or “*” instead of “X” to indicate a stop codon, and (d) the use of “X” to indicate “any amino acid,” as specified in the International Union of Pure and Applied Chemistry recommendations for amino acids nomenclature (International Union of Pure and Applied Chemistry, 1983).

These guidelines have been widely accepted in molecular diagnostics. Thus, they have been adopted by the Consensus recommendations of the College of American Pathologists (Gulley et al., 2007), the American College of Medical Genetics and Genomics, and the Association for Molecular Pathology (Richards et al., 2015). Public databases for germline (e.g., ClinVar) and cancer (e.g., COSMIC) variants have implemented the guidelines as well.

Currently, several tools for variant reporting which comply with HGVS nomenclature are available to guide laboratories. These may include public applications, such as Mutalyzer (Taschner & Den Dunnen, 2011) or VariantValidator (Freeman, Hart, Gretton, Brookes, & Dalgleish, 2018). HGVS software packages that run on Python (Wang et al., 2018) can also be used to manipulate sequence variants according to the nomenclature guidelines. Recently, a significant inconsistency in HGVS nomenclature was observed when comparing variant representations by three tools (SnpEff, Variant Effect Predictor, and Variation Reporter) that generate transcript and protein‐based variant nomenclature (Yen et al., 2017). Moreover, discordances were also noted between annotations generated by Snpeff and Variant Effect Predictor, and those in major germline and cancer databases such as ClinVar and COSMIC.

Besides variant descriptions, HGVS advises to include a reference sequence to unambiguously relate the variant to the used coding sequence (Den Dunnen et al., 2016). These sequences are preferably locus reference genome (LRG) sequences. In case an LRG is not available or “pending,” a RefSeq sequence is recommended (Macarthur et al., 2014; O'Leary et al., 2016). This is exemplified by a coding DNA transcript reference sequence (NM) frequently applied in routine practice for the detection of somatic variants in *EGFR, KRAS*, or *NRAS*. When reporting RefSeq sequences, the inclusion of a version number is important, as variants may refer to different genomic positions between reference sequences and between version numbers within one sequence.

External quality assessment (EQA) aims to monitor laboratory performance, compare results to international peers, and provide individual feedback to laboratories. Initial EQA schemes revealed a need to improve the consistent use of the appropriate nomenclature (Mueller, 2004; Touitou et al., 2009). For instance, assessment of reporting of cystic fibrosis transmembrane conductance regulator (*CFTR)* variants revealed that only 22% of the reports (*n* = 631) contained nomenclature conforming to HGVS recommendations, and 5% included nomenclature with the potential to generate interpretation errors in a clinical setting (Berwouts et al., 2011). Moreover, similar findings have also been reported for other indications, such as *BRCA1* and *BRCA2* analysis in breast cancer (Mueller, 2004), and hereditary recurrent fever (Touitou et al., 2009).

For predictive testing in non‐small cell lung cancer (NSCLC), several European providers assess HGVS compliance with regard to variant descriptions and reference sequences in EQA schemes. A first comparative study between providers (Tack, Deans, Wolstenholme, Patton, & Dequeker, 2016) suggested that higher HGVS compliance for epidermal growth factor receptor (*EGFR*) variants was correlated with the duration of operation of the scheme. It was therefore suggested that additional analyses are necessary to investigate whether a learning effect could be driving this improvement. Another study of EQA data in 2016 revealed highly variable descriptions of *EGFR* variants, and a lack of using tools to verify the variant descriptions. It was suggested that education is likely to be the way forward to eliminate the observed variability in data reporting (Deans, Fairley, Den Dunnen, & Clark, 2016).

This manuscript evaluates HGVS compliance over time. This is the first study in predictive testing for NSCLC and metastatic colorectal cancer (mCRC) to include this extent of longitudinal scheme data, as well as KRAS proto‐oncogene GTPase (*KRAS*) and NRAS proto‐oncogene GTPase (*NRAS*) variants besides *EGFR* variants. This approach allows for an evaluation if continued education has indeed exerted a positive effect since 2016 as previously suggested. Additional information was collected in the course of the EQA schemes, which enabled us to investigate the effect of continued EQA participation, as well as of the used test method as contributing factor, and their efficacy in overcoming previously reported residual discrepancies in HGVS compliance.

## 2 MATERIALS AND METHODS

The European Society of Pathology (ESP) and Gen&Tiss consortium organize yearly EQA schemes for variant analysis in *EGFR* for NSCLC, and *KRAS/NRAS* analysis for mCRC. Both schemes were organized according to the guideline on the requirements of EQA programs in molecular pathology (van Krieken et al., 2013) and the International Organization for Standardization (ISO) 17043 guideline for conformity assessment of proficiency testing (International Organization for Standardisation, 2010). Detailed organization of the EQA schemes has been previously described (Dequeker et al., 2016; Keppens et al., 2018; Tack et al., 2015). Participants were asked to analyze a set of formalin‐fixed tissue samples by their routine procedures. Then, they completed an electronic datasheet about their test results and the applied methods. The participants also submitted a written report with the molecular test result and interpretation, mimicking their routine practice.

Compliance with HGVS recommendations was scored from the written reports for the ESP EQA schemes, and from the electronic datasheets for the Gen&Tiss EQA schemes. Data from 351 international participants engaging in EQA schemes between 2012 and 2018 were included. The results and interpretation sections of the report were scored. If neither of these sections were available, nomenclature was scored from the provided list of tested variants. For Gen&Tiss, data from 64 French laboratories were scored from EQA schemes run between 2013 and 2018, from entries in an open text field in the electronic results sheet.

All variants in the *EGFR* (NSCLC), *KRAS*, and *NRAS* (mCRC) genes were validated before sample distribution by a reference laboratory. For evaluation of the variant descriptions according to HGVS, a team of international experts in molecular pathology agreed upon a predefined set of scoring criteria, based on HGVS key‐points as presented in Table 1. These criteria have been harmonized between schemes and were already described in more detail in the paper by Tack et al. (2016). When necessary, results were rechecked by the Alamut software used by the respective reference laboratory or Mutalyzer. After reaching an expert consensus result, outcomes of the scheme were communicated to the participants in an individual report.

**Table 1:** Overview of important HGVS key‐points and examples of errors observed in this study

	Variant nomenclature			Reference sequences		
	Key‐points (den Dunnen et al., 2016)					
	■ Nomenclature should always include the nucleotide level and protein level with inclusion of their respective prefixes 'c.' and 'p.' ■ Predicted consequences, that is, protein changes without experimental evidence (no RNA or protein sequence analyzed), should be given in parentheses since 2016 ■ Both three‐ (preferred) and one‐letter amino acid code may be used ■ For all descriptions the most 3′ position possible of the reference sequence is arbitrarily assigned to have been changed ■ For deletions, insertions, deletions‐insertions and duplications, the most 5′and most 3′ positions should be mentioned, and separated by an underscore on nucleotide and protein level, followed by their respective prefix: del, ins, delins, or dup ■ Specification of the nucleotide(s) or amino acid(s) (but not the number) is optional for deletions and duplications, but mandatory for insertions and deletion‐insertions ■ When the exact protein change is unknown, “X” or "Xaa" is used to indicate “any amino acid"			■ For diagnostic applications HGVS strongly recommends the Locus Reference Genomic sequence (LRG) ■ When no LRG is available or the LRG is pending, a RefSeq sequence, with its version (RefSeqGene or transcript) is recommended		
Gene	Variant description according to HGVS	Example of errors observed in EQA scheme	Explanation	Reference sequence according to HGVS^a^	Example of errors observed in EQA scheme	Explanation
*EGFR* (NM_005228.5)	c.2253_2276del p.(Ser752_Ile759del)	c.2253_2276del p.(Ser752_Ile759del15)	Inclusion of number of deleted number of amino acids	NM_005228.5	NM_005228	No version number
		c.2253–2276del p.(Ser752_Ile759del)	Use of ‐ instead of _ between positions			
	c.2237_2255delinsT p.(Glu746_Ser752delinsVal)	c.2237_2255delinsT p.Glu746_Ser752delinsVal	Omission of brackets at protein level	LRG_304t1	LRG_34	Outdated version number
		c.2237_2255>T p.Glu746_Ser752>Val	Usage of '>' instead of 'delins'			
*KRAS* (NM_033360.4)	c.34G>A p.(Gly12Ser)	34G>A p.(Gly12Ser)	Omission of 'c.' prefix at nucleotide level	NM_033360.4	NM_33360.3	Incorrect reference sequence
		c.34G>A Gly12Ser	Omission of 'p.' prefix at protein level and brackets			
	c.437C>T p.(Ala146Val)	c.437C>T p. Ala146 Val	Inclusion of additional spaces	NM_004985.4	GRCh38	Other format^b^
		c437C>T p(Ala146Val)	Omission of '.' after prefix at any level			
*NRAS* (NM_002524.5)	c.35G>A p.(G12D)	c.35g>a p.(Gly12Asp)	Nucleotides written in small letters	LRG_92t1	NG_007572.1	Other format^b^
		G12D	Use of traditional nomenclature			
	c.183A>T p.(Gln61His)	p.(Gln61His)	Only protein level given	NM_002524.5	ENSG00000213281	Other format^b^
		c.183A>T	Only nucleotide level given			

*Note*: The reference sequences for the description of variants in this study were *EGFR* NM_005228.5, *KRAS* NM_033360.4, and *NRAS* NM_002524.5.

Abbreviations: *EGFR*, epidermal growth factor receptor; EQA, external quality assessment; HGVS, Human Genome Variation Society; *KRAS*, KRAS proto‐oncogene GTPase; LRG, locus reference genomic sequence; *NRAS*, NRAS proto‐oncogene GTPase.

^a^ Example, multiple correct reference sequences are available for these genes.

^b^ Other format besides NM or LRG, but still correct reference sequence.

Nomenclature scores obtained during the schemes were then re‐evaluated, to ensure harmonized scoring between the two providers and all scheme years. HGVS compliance was evaluated only for cases that included a variant, but not for false‐positive results in wild‐type cases. Cases without nomenclature were also excluded, for example, in case of a technical failure, false‐negative result, or descriptive sentences in the form of “we detected a variant.” If a case comprised multiple variants, all variants were evaluated separately. Entries were then classified into “correct” and “incorrect” categories. Correct nomenclature according to the EQA scoring criteria, was defined as all variants reported in complete accordance with the HGVS guidelines. For incorrect nomenclature, a further division was made between (a) “type 1” small clerical errors (such as the omission of brackets or stop marks, spaces, etc.), (b) “type 2” errors with potential therapeutic impact (e.g., incorrect amino acid abbreviations, or using traditional “legacy” nomenclature), and (c) “type 3” errors, that include only reporting the variants at protein or nucleotide level. Traditional nomenclature (e.g., T790M) was considered an error because of the absence of the nucleotide description and “p.” prefix, however, a correct description with a one‐letter amino acid abbreviation (e.g., c.2369C>T p.(T790M)) was considered correct.

Inclusion of the reference sequences with a version number was evaluated in the diagnostic report for both providers. Reference sequences which were correct according to the EQA scoring criteria, included the recommended LRG, or NM format with inclusion of the current version number at the time of assessment. Laboratories with an outdated version number were not penalized, but received a suggestive comment to raise awareness of the availability of a more recent version number.

Results were analyzed on two levels. First, the percentage of HGVS compliant nomenclature for each of the variants is separately provided. Secondly, HGVS compliance is shown on laboratory level, to evaluate the performance (a) over time, (b) relative to the number of EQA participations, and (c) in relation to the applied test methodology. Statistics were performed by SPSS v25.0 (IBM, Armonk, NY). *χ* ^2^ tests for contingency tables were applied, and Fisher's Exact test was used for cell counts below five. All significance levels were set at *α* = 0.05 with Bonferroni correction for multiple comparisons. The applied reference sequences for the description of the variants in this study were *EGFR* NM_005228.5, *KRAS* NM_033360.4, and *NRAS* NM_002524.5.

## 3 RESULTS

### 3.1 Performance of different variants

In Table 1, we present an overview of the most important recommendations for variant reporting in NSCLC and mCRC, as well as examples of noncompliance with HGVS as observed in this study.

In total, 4,802 variant entries were evaluated during the ESP and Gen&Tiss EQA schemes combined, of which 749 (15.6%) did not include nomenclature (e.g., written in the form of “a variant was detected”). This resulted in 4,053 assessed variants, of which 12.1% was classified as complying with HGVS recommendations. There was no significant difference in the degree of compliance between both EQA providers (15.4%, *n* = 2,530 for ESP and 9.3%, *n* = 1,523 for Gen&Tiss; data not shown).

There was a large variability in the percentage of nomenclature reported in complete accordance with HGVS, displaying a wide range between the different variants. However, overall, there were no significant differences in the degree of HGVS compliance for reporting of variants in the *EGFR* (12.0%, *n* = 2,377), *KRAS* (12.4%, *n* = 1,217), and *NRAS* genes (11.9%, *n* = 459; Table S1).

In total, 64.4% (*n* = 4,053) of errors consisted of small (type 1) clerical errors like inclusion of a space, omission of a stop mark, or the absence or incorrect use of brackets on protein level. In 2.4% of entries, a larger (type 2) error was observed for which the “c.” or “p.” prefix on the nucleotide or protein level was omitted, traditional “legacy” nomenclature (e.g., T790M) was used, or an incorrect amino acid code was given (e.g., Tyr instead of Thr). For 2.0% of entries, the variant was described only on nucleotide or protein level (type 3). In 19.1% a combination of the abovementioned errors was made.

Errors with a potential therapeutic impact, for example, type 2 and type 3 errors or a combination, were observed more frequently for variants in the *EGFR* gene (35.6%, *n* = 2,377) compared with variants detected in the *KRAS* (18.1%, *n* = 1,217) or *NRAS* (16.8%, *n* = 456) genes (Table S1).

Table 2 displays only those variants that were repeatedly distributed in successive EQA schemes. An increase in the percentage of HGVS compliance was observed for all variants between the first and last scheme of inclusion.

**Table 2:** Percentage of entries with correct nomenclature for returning variants in subsequent EQA schemes

Scheme year		2012	2013	2014	2015	2016	2017	2018
Gene	Variant	% HGVS compliant nomenclature (*N*)						
*EGFR* (NM_005228.5)	c.2155G>A p.(Gly719Ser)	/	/	3.8 (131)	/	6.3 (32)	/	/
	c.2155G>T p.(Gly719Cys)	/	0.0 (37)	/	9.1 (44)	/	/	/
	c.2235_2249del p.(Glu746_Ala750del)	/	/	/	4.7 (121)***	25.9 (27)	47.2 (36)***	/
	c.2236_2250del p.(Glu746_Ala750del)	/	/	5.3 (113)	/	/	/	20.0 (95)**
	c.2303G>T p.(Ser768Ile)	/	0.0 (17)	/	13.8 (29)	8.7 (23)	/	/
	c.2369C>T p.(Thr790Met)	/	7.0 (71)	4.3 (93)**	5.2 (97)*	18.1 (83)	21.7 (92)**	16.5 (194)
	c.2573T>G p.(Leu858Arg)	/	3.0 (168)***	0.0 (85)***	6.6 (150)**	18.0 (130)	38.6 (133)***	27.1 (96)***
*KRAS* (NM_033360.4)	c.35G>A p.(Gly12Asp)	/	3.4 (167)	/	/	/	45.8 (48)***	/
	c.38G>A p.(Gly13Asp)	3.2 (93)	0.0 (147)***	10.6 (104)***	/	/	/	/
	c.183A>C p.(Gln61His)	/	/	/	8.3 (48)	/	39.6 (48)***	/
	c.183A>T p.(Gln61His)	/	/	/	/	17.0 (47)	/	33.9 (109)*
*NRAS* (NM_002524.5)	c.181C>A p.(Gln61Lys)	/	6.3 (111)**	/	8.4 (95)	27.3 (77)***	/	/

*Note*: In case a variant was distributed in both ESP and Gen&Tiss EQA schemes within a specific year, average percentages for both schemes were presented. The reference sequences for the description of the variants in this study were *EGFR* NM_005228.5, *KRAS* NM_033360.4, and *NRAS* NM_002524.5.

/: Variant not distributed during this scheme year. Asterisks represent a statistical difference compared with other scheme years. *χ* ^2^ test or Fisher's Exact test for cell counts below 5.

Abbreviations: *EGFR*, epidermal growth factor receptor; EQA, external quality assessment; ESP, European Society of Pathology; HGVS, Human Genome Variation Society; *KRAS*, KRAS proto‐oncogene GTPase; *NRAS*, NRAS proto‐oncogene GTPase; *N*, number of entries scored.

* *p* < .05.

** *p* < .01.

*** *p* < .001.

### 3.2 Performance by time and EQA participation

On laboratory level, the percentage of HGVS compliance improved significantly (*p* < .001) over time from 2.0% (*n* = 94) in 2012 to 22.3% (*n* = 206) in 2018 (Table 3). An improvement was also observed after multiple EQA scheme participations (*p* < .001). Thus, 4.6% of cases (*n* = 614) were reported according to HGVS recommendations during a laboratory's first participation, compared to 19.5% of cases when laboratories had participated six times (*n* = 41; Table 3). Laboratories who reported variants in complete accordance with HGVS guidelines were significantly less likely (*p* < .001) to make an error in HGVS nomenclature in their subsequent participation (58.0% compliance compared with 11.6% for laboratories with non‐HGVS compliance; Table 3).

**Table 3:** Percentage of HGVS compliant nomenclature and reference sequences in relation to scheme year, number of participations and performance in the previous scheme

EQA provider	ESP		Gen&Tiss		Total
Indication	NSCLC	mCRC	NSCLC	mCRC	NSCLC + mCRC
**% HGVS compliant nomenclature (*N*)**					
**Time**					
2012	/	2.0 (94)***	/	/	2.0 (94)***
2013	5.8 (86)	5.7 (122)***	0.0 (44)**	0.0 (49)**	4.0 (301)***
2014	2.2 (139)**	10.6 (104)*	0.0 (44)**	0.0 (47)**	4.2 (334)***
2015	1.8 (110)*	/	4.4 (46)	8.3 (48)	3.9 (204)***
2016	11.8 (93)	24.6 (122)*	12.2 (41)	14.3 (49)	17.4 (305)**
2017	15.2 (92)**	27.9 (86)**	40.5 (42)***	39.6 (48)***	27.6 (268)***
2018	9.3 (97)	33.9 (109)***	/	/	22.3 (206)***
**Number of EQA participations**					
1	3.1 (258)***	8.3 (240)***	0.0 (55)**	0.0 (61)***	4.6 (614)***
2	7.8 (141)	14.5 (145)	0.0 (48)**	0.0 (54)***	8.3 (388)**
3	6.4 (94)	29.0 (107)***	7.0 (43)	10.4 (48)	15.4 (292)
4	11.8 (68)	24.7 (73)	15.0 (40)	20.5 (44)	18.2 (225)**
5	28.2 (39)***	27.1 (48)	48.4 (31)***	47.1 (34)***	36.2 (152)***
6	0.0 (17)	33.3 (24)*	/	/	19.5 (41)
**Performance between two participations**					
Correct in previous scheme	16.0 (25)	79.6 (49)***	60.0 (5)*	55.6 (9)**	58.0 (88)***
Error in previous scheme	8.2 (280)	12.3 (285)	13.6 (147)	14.6 (164)	11.6 (876)
**% HGVS compliant reference sequences (*N*)**					
**Time**					
2012	/	16.7 (102)	/	/	16.7 (102)***
2013	28.6 (105)	27.5 (131)	44.4 (45)	46.9 (49)	33.1 (330)
2014	24.1 (141)*	11.3 (124)**	54.6 (44)	55.3 (47)	27.5 (356)*
2015	36.6 (112)	/	56.5 (46)	14.6 (48)***	35.9 (206)
2016	43.2 (95)*	23.8 (122)	38.1 (42)	40.4 (47)	34.3 (306)
2017	31.5 (92)	22.1 (104)	45.2 (42)	60.4 (48)**	35.0 (286)
2018	33.0 (97)	33.1 (109)**	/	/	33.0 (206)
**Number of EQA participations**					
1	25.0 (268)***	15.2 (250)***	43.9 (57)	46.8 (62)	25.0 (637)***
2	31.1 (148)	29.1 (158)*	61.2 (49)*	50.9 (53)	36.5 (408)*
3	41.2 (97)*	20.0 (115)	54.8 (42)	19.2 (47)***	31.6 (301)
4	43.5 (69)*	25.3 (83)	36.6 (41)	44.2 (43)	36.1 (236)
5	40.0 (40)	26.4 (53)	40.0 (30)	58.8 (34)	39.5 (157)*
6	40.0 (20)	39.4 (33)*	/	/	39.6 (53)
**Performance between two participations**					
Correct in previous scheme	65.8 (114)***	59.1 (88)***	72.4 (76)***	57.4 (68)***	63.9 (346)***
Error in previous scheme	27.6 (203)	19.0 (300)	26.9 (78)	31.4 (102)	24.3 (683)

*Note*: Percentages are calculated on laboratory level. No EQA scheme was organized in 2015. Analyses only include entries for which corresponding method information was available. The number of scored entries differed for nomenclature versus reference sequence analysis given that for some participants no nomenclature was present on the written reports. HGVS compliant nomenclature was defined as all entries written according to HGVS format. HGVS compliant reference sequences included sequences in LRG or NM format with inclusion of a version number. The number of EQA participations (1–6) reflected the number of times an individual laboratory participated to successive EQA scheme years. Asterisks represent a significant difference compared to other categories by *χ* ^2^ test or Fisher's exact test for cell counts below 5.

Abbreviations: EQA, external quality assessment; ESP, European Society of Pathology; HGVS, Human Genome Variation Society; *N*, number of entries scored; NSCLC, non‐small cell lung cancer; mCRC, metastatic colorectal cancer.

* *p* < .05.

** *p* < .01.

*** *p* < .001; /, not applicable.

In the total reference sequences assessed in the written reports (*n* = 1,792), 31.9% of participants reported a reference sequence in the LRG or NM format with the most recent version number. In 9.5% of entries, a reference was included with a previous, but correct, version number. For 3.1%, the reference sequence was written in a format (e.g., NC, NG, or ENST sequences) that was not recommended by the EQA provider. For 10.0% of entries, no version number was added. An error in the reference sequence was observed in 1.8% of entries, while in 43.8% of cases no reference sequence was reported at all (Table S2).

The percentage of participants who reported a correct reference sequence, increased significantly (*p* < .001) over time from 16.7% in 2012 (*n* = 102) to 33.0% in 2018 (*n* = 206; Table 3). After six EQA participations, 39.6% (*n* = 637) of participants included a HGVS compliant reference sequence compared to 25.0% of first time participants (*n* = 53), which is a significant increase (*p* < .001). Participants that reported an appropriate sequence were significantly (*p* < .001) more likely to do so again in the next EQA participation.

### 3.3 Influence of testing methods

**Figure 1:**
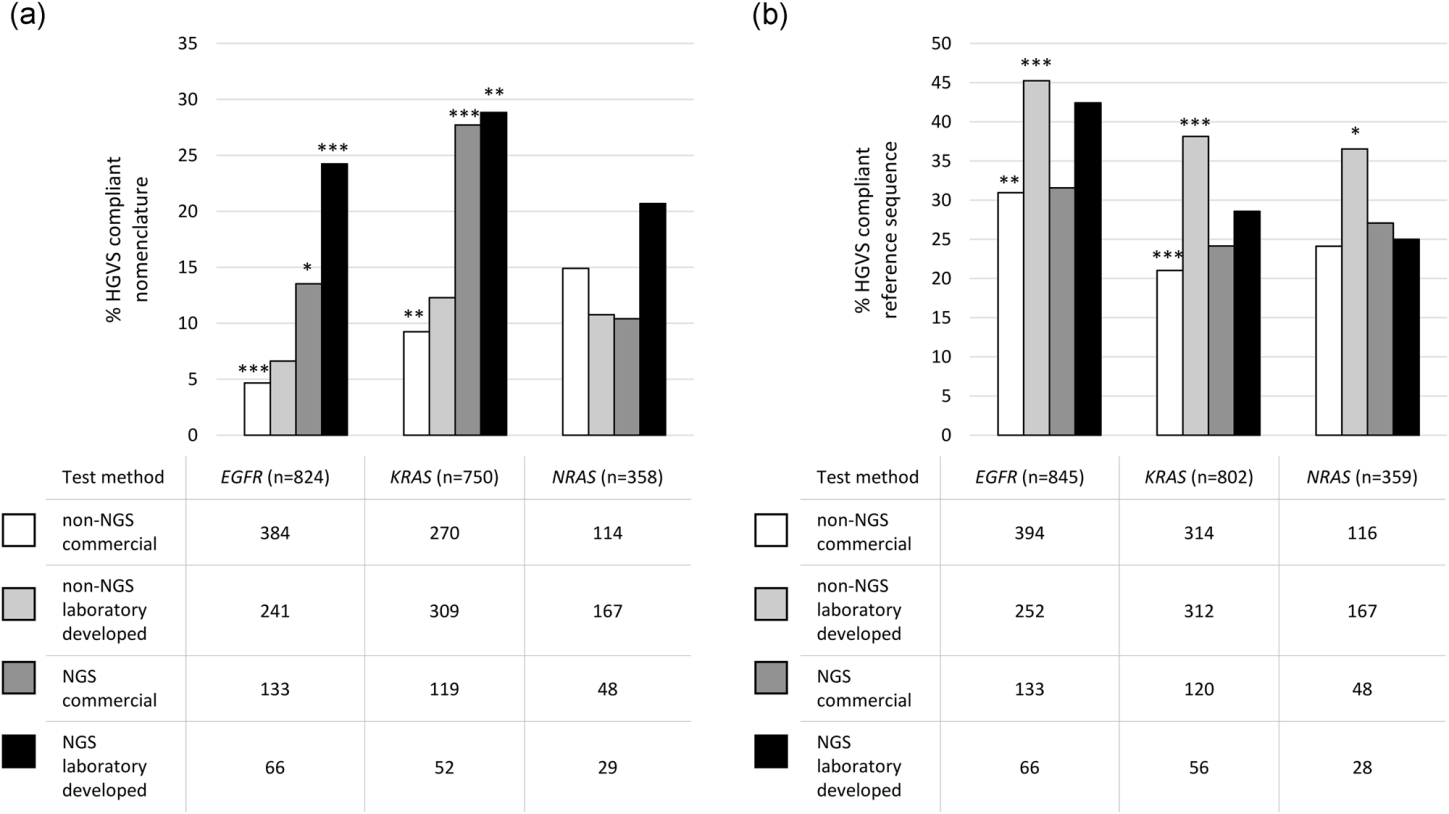
Percentage of HGVS compliant nomenclature (a) and reference sequences (b) related to used analysis techniques for the detection of *EGFR, KRAS*, and *NRAS* variants. Analyses only included entries for which corresponding method information was available. The number of scored entries differed for nomenclature versus reference sequence analysis given that for some participants no nomenclature was present on the written reports. HGVS compliant nomenclature was defined as all entries written according to HGVS format. HGVS compliant reference sequences included were those in LRG or NM format with inclusion of a version number. Noncommercial methods include fragment analysis, dideoxy or Sanger sequencing. An overview of commercial kits can be found in Table S3. Asterisks represent significant differences compared to other test methods by χ^2^ tests, *p < .05, **p < .01, ***p < .001. Abbreviations: *EGFR*, epidermal growth factor receptor; HGVS, Human Genome Variation Society; *KRAS*, KRAS proto‐oncogene GTPase; NGS, next‐generation sequencing; *NRAS*, NRAS proto‐oncogene GTPase

The reporting of variant nomenclature according to HGVS recommendations differs depending on the testing methodology used. In total, 9.6% and 9.9% of HGVS compliant entries were observed for users of a commercial (*n* = 768) or a noncommercial method (*n* = 717), respectively (both non‐next‐generation sequencing (NGS) based methods) (Figure 1a). Laboratories using NGS (*n* = 447) complied with the recommendations in 20.9% of assessed variants. Compliance was observed more frequently when applying noncommercial NGS panels compared with commercial NGS panels (24.6% *n* = 300 vs. 17.2%, *n* = 147). The difference between commercial and noncommercial methods was significant (*p* < .001) for *EGFR* and *KRAS* testing, but not for *NRAS* analysis (*p* = .385). An overview of the commercial test kits used by the laboratories in this study and the nomenclature used to describe the variants in the package inserts is given in Table S3.

HGVS compliance for the reference sequences was higher for laboratories using noncommercial non‐NGS testing methods (40.0%, *n* = 731) and noncommercial NGS panels (32.0%, *n* = 150) compared with laboratories using commercial non‐NGS tests (25.4%, *n* = 824) and commercial NGS panels (27.6%, *n* = 301; Figure 1b).

## 4 DISCUSSION

The aim of the ESP and Gen&Tiss EQA schemes for predictive biomarker testing in NSCLC and mCRC is to support diagnostic laboratories in the correct detection and reporting of molecular variants.

The aim of this study was to evaluate the effect of EQA in improving the previously reported inconsistencies in complying with HGVS nomenclature. Previous studies focused on data from one or two consecutive EQA schemes for the reporting of *EGFR* variants in NSCLC. To our knowledge, this is the first study to include data from at least five successive EQA schemes, that includes variants important for therapy selection in mCRC and NSCLC.

In particular, both the studies of Tack et al. (2016) and Deans et al. (2016) reported an improvement in HGVS compliance between EQA scheme distributions. It was hypothesized that this improvement was due to the positive influence of EQA feedback, but additional data was needed to confirm interpretations about the residual inconsistencies.

These results derived from a larger data set now confirm previous findings, that is, variant descriptions are highly variable between (a) the participants, (b) the assessed genes of interest, and (c) the different variants within one gene. However, we were able to demonstrate that EQA participation contributes to a large extent to the improvement observed over time as well. Because of the collection of additional laboratory information, we could reveal that residual inconsistencies may depend on the complexity of the variant itself, and especially on the applied testing method by the EQA participants. This suggests that continuous education is a valuable tool, but might in itself not be sufficient to reach a harmonized way of reporting throughout Europe.

More specifically, we observed an improvement over time for HGVS compliance of variant nomenclature and reference sequences. This suggests that laboratories are successfully adopting the updated recommendations published in 2016 (den Dunnen et al., 2016). As this improvement coincided with additional EQA participations, participants are presumably using the individual feedback provided at the end of a scheme. Nevertheless, many inconsistencies remained in the latest EQA scheme of 2018. There were more serious errors (errors that differ from small clerical errors) observed for *EGFR* analysis, compared with *KRAS* and *NRAS* analysis. One explanation could be that many *EGFR* variants included in the schemes were complex deletions/insertions compared with single nucleotide variants for the *KRAS* or *NRAS* genes. Our analysis also indicated that laboratories who did not comply with HGVS were less likely to comply with HGVS nomenclature in their next participation. This suggests that learning to adopt HGVS recommendations takes time. Annotation and alignment algorithms might not be updated if they are incorporated in the laboratory information system.

Second, our findings reveal that the analytical test method was related to the reported variant description. This might further contribute to the difference observed between NSCLC and mCRC, as more EQA participants used commercial test kits to analyze *EGFR* variants compared to *KRAS/NRAS* variants. Therefore, we evaluated the nomenclature used in the different package inserts from commercially available kits. Remarkably, the majority of these inserts did not comply with HGVS recommendations, even those that are CE‐ or CE‐IVD labeled (Directive/79/EC, 1998). With the increasing number and complexity of variants analyzed by NGS, user experience with software and annotation tools are an important factor to consider when interpreting these data.

Even though many useful databases and tools exist, these guidance systems should be thoroughly validated before implementation into clinical practice. With the increasing importance of NGS, the use of variant annotation software will become more important in reporting molecular results. This is emphasized by the paper of Yen et al. (2017), who showed substantial discordance between annotation tools and databases in the description of variants, especially of insertions and/or deletions. In addition, not all available tools are considered sufficiently adequate to validate HGVS descriptions. Many journals, such as Human Mutation, now request validation of variant descriptions according to HGVS recommendations before submission, by for instance Mutalyzer, VariantValidator, or Alamut (den Dunnen, 2019).

Even though we observed a higher proportion of “type 2” errors for complex mutations, it remains to be elucidated how many of these include an infringement on the 3′ rule (which is defined as: ‘for all descriptions the most 3′ position possible of the reference sequence should be assigned to indicate a change’) by annotation tools.

Nevertheless, even when applying a commercial test kit, it is the final responsibility of the laboratory to comply with HGVS nomenclature when reporting the test results. Awareness and education of the laboratory staff are important at multiple levels, as predictive testing entails a cooperation of molecular biologists, clinical scientists in molecular pathology, and pathologists. First, in our experience, laboratories often apply traditional “legacy” nomenclature (such as “T790M” or “L858R”) to cater for the needs of clinicians interpreting the clinical reports. However, clinicians need to be aware of the updated nomenclature guidelines whether or not accompanied by traditional nomenclature. Misinterpretation of test results may lead to the incorrect inclusion of patients in, or exclusion of patients from clinical trials, selected by variants submitted to databases. Second, several non‐NGS commercial kits for *EGFR* or *RAS* testing currently do not discriminate between the exact nucleotide changes that are possible. Instead, they report the presence of an “exon 19 deletion” or “codon 12 mutation,” leaving laboratories reliant on their own experience with regard to nomenclature. Hence it is not surprising that 15.9% (*n* = 4,802) of entries did not include assessable variant descriptions.

EQA providers have the possibility to detect systematic shortcomings in variant detection methods based on the availability of data from laboratories worldwide. In this case, EQA providers report back to the method manufacturers after which appropriate actions are taken to improve the methodology. These data now suggests that besides information on variant identification, a holistic collaboration between EQA providers and companies providing kits and/or software solutions might be useful to further harmonize nomenclature. Moreover, EQA providers are advised to request additional information on the annotation tools used during the course of the EQA scheme. A recent follow‐up of EQA results in clinical laboratories revealed that participants frequently contact the company in case of unexplained false‐negative or false‐positive results (unpublished data). Laboratories are advised to also establish contact with the company in case of discrepancies in reported nomenclature, and can be assisted by the EQA provider if necessary.

Besides nomenclature, EQA scheme participation positively affected the inclusion of an appropriate reference sequence as shown in this study. However, the effect of continued EQA participation was smaller compared to the reporting of variants. Laboratories who included a correct sequence with a previous version number were not penalized, but did receive a suggestive comment mentioning the availability of a more recent version number. Of more concern is the large fraction of entries for which no version number was included, similar to previously reported results (Tack et al., 2016). Inclusion of a version number is of utmost importance. Providing a database accession number without version number is not sufficient to identify a sequence in the database unambiguously and several different versions may exist for any given accession. In most cases only the annotation changes, while the sequence remains unaltered, but this is not always the case. One example is that due to an update of the reference sequence (from NM_004333.1 to NM_004333.4) for the *BRAF* gene, the c.1796T>A p.(Val599Glu) variant was changed to c.1799T>A p.(Val600Glu; Tack et al., 2016). Both laboratories and EQA providers are thus advised to monitor new developments in the recommendations so that they can rapidly introduce these changes in routine practice. Currently, EQA providers are already reviewing the current guidelines before the start of an EQA scheme. Similar to variant descriptions, method dependency was observed for reporting of reference sequences. HGVS compliance was higher for laboratories using noncommercial methods compared with those using commercial CE‐ or CE‐IVD labeled kits (both NGS and non‐NGS based methods). Increased experience might play an important role, as laboratories using noncommercial methods need to setup and validate their methods, giving them a certain know‐how that allows them to select the appropriate reference sequence themselves, rather than relying on those reported by software packages for commercial techniques. In addition, non‐NGS based commercial tests often are integrative systems, for which the users do not need to compare the variant to a specific reference sequence.

Finally, these data do not provide information with regard to the clinical interpretation of the variants by the treating physician and the consequences for treatment strategy remain to be elucidated. In this study, the majority of errors consisted of small “type 1” clerical errors, which may be easier to resolve. The most frequent example was the omission of brackets, which is recommended by HGVS since 2016 for prediction of the expected amino acid change. Even though the risk for misinterpretation might be negligible, small differences could result in incomplete search queries or incorrect cross‐referencing between databases for various purposes.

Besides the variants included in this study, nomenclature of predictive variants in novel cancer‐related genes might be even more complex. For instance, *MET* gene variants associated with exon 14 skipping in NSCLC usually consist of complex deletions also affecting intron sequences (e.g., NM_001127500.3: c.3077_3082+9del p.[Leu982_Asp1028]; Frampton et al., 2015). Another example includes *BRCA1/2* variants as predictive markers in ovarian cancer (Weren et al., 2017). These stress the importance of complying with recommendations for treatment decisions and unambiguous registration in clinical databases.

To conclude, even though recommendations for reporting variants have been around since 2000, inconsistencies in HGVS compliance in the EQA schemes remain. Participation in EQA contributes to a large extent to the improvement over time. However, the significant association between two instances of participation suggests that laboratories do not readily update their reporting strategy, especially for reference sequences. These findings illustrate that the test method is an important influencing factor. A multidisciplinary collaboration between laboratories, EQA providers and manufacturers of kits and software solutions might aid the harmonization of nomenclature. EQA providers could assist nomenclature policy makers by providing insight into commonly occurring problems worldwide. Moreover, EQA providers as well as laboratories are advised to monitor changes in recommendations and anticipate nomenclature assessment for upcoming markers which might be even more complex.

## FUNDING

An unrestricted research grant from Pfizer Oncology was received by EMCD for the organization of the ESP Lung EQA schemes, irrespective of the research performed during this study.

## CONFLICT OF INTERESTS

C. K., V. T., and K. D. have nothing to declare. E. R. participated in advisory boards for Roche, Bristol‐Myers Squibb, and AstraZeneca. M. J. L. L. is consultant in advisory boards for AstraZeneca, Bayer, Janssen Pharmaceuticals, Merck, Nimagen, Roche, and received financial support from AstraZeneca and Bristol‐Myers Squibb (all fees to the institute). E. S. performed lectures for Illumina, Novartis, Pfizer, BioCartis; is consultant in advisory boards for AstraZeneca, Pfizer, Novartis, BioCartis; and received financial support from Roche, Biocartis, Bristol‐Myers Squibb, and Pfizer (all fees to the institute). E. M. C. D. received an unrestricted research grant from Pfizer Oncology to support the organization of the ESP Lung EQA schemes.

## AUTHOR CONTRIBUTIONS

C. K. (0000–0002‐4498–8386), V. T. (0000–0001‐8464–9541), and E. M. C. D. conceived and designed the study setup. C. K., V. T., K. D. (0000–0002‐0418‐587X), E. R. (0000–0002‐9046‐257X), and E. M. C. D. collected the results in accordance to ISO 17043. E. R., M. J. L. L. (0000–0003‐1290–1474), and E. S. (0000–0003‐3655‐143X) were responsible for technical expertise during the EQA schemes and conceiving of the nomenclature categories during the scheme. C. K., V. T., K. D., and E. M. C. D. interpreted the data. C. K., E. S., and E. M. C. D. were responsible for harmonizing the data, statistical analysis, and writing of the manuscript. All authors critically revised the manuscript for important intellectual content.

## ETHICAL STATEMENT

The samples originated from tissue blocks of leftover patient material obtained during routine care, and were excluded from informed consent. The molecular testing performed in the schemes reflected the routine tests prescribed, as no additional molecular testing was performed. In addition, each scheme organizer signed a subcontractor agreement stating that the way in which the samples were obtained was conform national legal requirements for the use of patient samples from their biobank.

## Supporting information

Supporting information

## Data Availability

The datasets generated and/or analyzed during the current study are available from the corresponding author upon reasonable request.
