# Supplementary material for: Variation in nomenclature of somatic variants for selection of oncological therapies: Can we reach a consensus soon?
Source: Hum Mutat. 2019 Oct 14;41(1):7–16. doi: 10.1002/humu.23926 (PMC6973115; doi:10.1002/humu.23926)
Supplement: Supplementary file 1 — Supporting information [file HUMU-41-7-s001.pdf]

Title Variation in nomenclature of somatic variants for selection of oncological therapies: can we reach a consensus soon?

Journal Human Mutation

Authors Cleo Keppens, Véronique Tack, Kelly Dufraing, Etienne Rouleau, Marjolijn J.L. Ligtenberg, Ed Schuuring, Elisabeth M.C. Dequeker

Correspondence Prof. Dr. Elisabeth M.C. Dequeker
University of Leuven
Department of Public Health and Primary Care
Biomedical Quality Assurance Research Unit
Kapucijnenvoer 35d Box 7001
3000 Leuven
Belgium
Tel: +3216 345881, E-mail: els.dequeker@kuleuven.be

Supp. Table S1. Assigned nomenclature categories for the different variants distributed during the ESP and Gen&Tiss EQA schemes for lung and colorectal cancer. Results were classified into four distinct categories: correct HGVS nomenclature used, type 1 errors without risk for misinterpretation, type 2 and 3 errors with possible implications for incorrect interpretation. Type 1 errors included small clerical errors like inclusion of a space, omission of stop mark, or absence or incorrect use of brackets on protein level. Type 2 errors consisted of the absence of a 'c.' or 'p.' prefix on the coding and protein levels, respectively, the use of traditional nomenclature (such as 'T790M' or 'L858R'), or adoption of an incorrect (one or three-letter) amino acid code. Type 3 errors included cases for which only the nucleotide or amino acid level was given but not both. In case of multiple errors within a case, a combination of error types was assigned as a separate category, e.g. only reporting the amino acid level (type 3), without the use of brackets (type 1). The reference sequences for the description of variants in this study were *EGFR* NM_005228.5, *KRAS* NM_033360.4 and *NRAS* NM_002524.5. Abbreviations: *EGFR*, Epidermal Growth Factor Receptor; EQA, External Quality Assessment; ESP, European Society of Pathology; *KRAS*, Kirsten ras oncogene homolog; *NRAS*, NRAS proto-oncogene GTPase; #, number.

Marker	Variant	EQA scheme	# entries scored	% correct nomenclature	% single type 1 error	% single type 2 error	% single type 3 error	% combination of error types	% *type 1+2*	% *type 1+3*	% *type 1+2+3*	% *type 2+3*
EGFR NM_005228.5 (Lung schemes)	c.2065_2067delinsTTT p.(Val689Phe)	Gen&Tiss 2013	10	0.0	60.0	0.0	0.0	40.0	*20.0*	*10.0*	*0.0*	*10.0*
	c.2126A>T p.(Glu709Val)	Gen&Tiss 2014	12	0.0	91.7	0.0	0.0	8.3	*0.0*	*8.3*	*0.0*	*0.0*
	c.2155G>A p.(Gly719Ser) (2x)	Total	163	4.3	42.3	4.3	0.0	49.1	*3.1*	*11.0*	*4.9*	*30.1*
		ESP 2014	131	3.8	33.6	4.6	0.0	58.0	*3.1*	*12.2*	*6.1*	*36.6*

		Gen&Tiss 2016	32	6.3	78.1	3.1	0.0	12.5	3.1	6.3	0.0	3.1
	c.2155G>T p.(Gly719Cys) (2x)	Total	81	4.9	76.5	2.5	2.5	13.6	0.0	4.9	1.2	7.4
		Gen&Tiss 2013	37	0.0	81.1	2.7	0.0	16.2	0.0	8.1	0.0	8.1
		Gen&Tiss 2015	44	9.1	72.7	2.3	4.5	11.4	0.0	2.3	2.3	6.8
	c.2156G>C p.(Gly719Ala)	Gen&Tiss 2013	39	0.0	84.6	0.0	0.0	15.4	0.0	7.7	0.0	7.7
	c.2235_2249del p.(Glu746_Ala750del) (4x)	Total	184	15.8	48.4	3.3	2.2	30.4	7.6	9.2	3.3	10.3
		ESP 2015	87	3.4	43.7	5.7	2.3	44.8	10.3	14.9	2.3	17.2
		ESP 2016	27	25.9	44.4	0.0	0.0	29.6	7.4	7.4	7.4	7.4
		Gen&Tiss 2015	34	5.9	76.5	2.9	2.9	11.8	0.0	5.9	0.0	5.9
		Gen&Tiss 2017	36	47.2	36.1	0.0	2.8	13.9	8.3	0.0	5.6	0.0
	c.2236_2248delinsCAAC p.(Glu746_Ala750delinsGlnPro)	ESP 2018	80	31.3	21.3	2.5	7.5	37.5	5.0	18.8	7.5	6.3
	c.2236_2250del p.(Glu746_Ala750del) (2x)	Total	208	12.0	31.3	2.4	5.8	48.6	6.3	16.3	6.7	19.2
		ESP 2014	113	5.3	41.6	2.7	0.9	49.6	8.8	13.3	5.3	22.1
		ESP 2018	95	20.0	18.9	2.1	11.6	47.4	3.2	20.0	8.4	15.8
	c.2237_2255delinsT p.(Glu746_Ser752delinsVal)	Gen&Tiss 2013	27	3.7	51.9	0.0	0.0	44.4	18.5	14.8	3.7	7.4
	c.2238_2256delinsG p.(Leu747_Ser752del)	Gen&Tiss 2013	24	4.2	70.8	0.0	0.0	25.0	12.5	4.2	4.2	4.2
	c.2240_2257del p.(Leu747_Pro753delinsSer)	ESP 2016	35	20.0	34.3	0.0	8.6	37.1	20.0	8.6	5.7	2.9
	c.2253_2276del p.(Ser752_Ile759del)	Gen&Tiss 2014	18	0.0	77.8	0.0	0.0	22.2	5.6	11.1	0.0	5.6
	c.2303G>T p.(Ser768Ile) (3x)	Total	69	5.8	71.0	2.9	1.4	15.9	4.3	4.3	0.0	7.2
		Gen&Tiss 2013	17	0.0	76.5	5.9	0.0	17.6	5.9	5.9	0.0	5.9
		Gen&Tiss 2015	29	13.8	72.4	0.0	3.4	10.3	3.4	0.0	0.0	6.9
		Gen&Tiss 2016	23	8.7	65.2	4.3	0.0	21.7	4.3	8.7	0.0	8.7
	c.2327G>A p.(Arg776His)	ESP 2014	35	14.3	45.7	0.0	0.0	40.0	0.0	8.6	11.4	20.0
	c.2369C>T p.(Thr790Met) (7x)	Total	630	12.9	27.3	4.6	7.0	48.3	6.3	14.3	10.0	17.6
		ESP 2013	71	7.0	31.0	2.8	0.0	59.2	2.8	21.1	12.7	22.5
		ESP 2014	93	4.3	39.8	3.2	1.1	51.6	2.2	8.6	10.8	30.1
		ESP 2015	97	5.2	33.0	3.1	5.2	53.6	17.5	7.2	3.1	25.8
		ESP 2016	83	18.1	30.1	1.2	2.4	48.2	9.6	16.9	7.2	14.5
		ESP 2017	92	21.7	35.9	13.0	6.5	22.8	9.8	4.3	5.4	3.3

		ESP 2018 (2x)	194	16.5	11.9	4.1	15.5	52.1	*1.0*	*21.6*	*15.5*	*13.9*
	c.2573T>G p.(Leu858Arg) (12x)	Total	762	14.8	50.9	3.4	3.4	27.4	*3.3*	*9.6*	*4.1*	*10.5*
		ESP 2013	83	6.0	33.7	4.8	0.0	55.4	*1.2*	*19.3*	*8.4*	*26.5*
		ESP 2015	104	6.7	42.3	2.9	1.9	46.2	*11.5*	*8.7*	*1.9*	*24.0*
		ESP 2016	89	21.3	44.9	3.4	2.2	28.1	*4.5*	*12.4*	*11.2*	*0.0*
		ESP 2017	91	29.7	35.2	9.9	5.5	19.8	*6.6*	*5.5*	*4.4*	*3.3*
		ESP 2018	96	27.1	18.8	3.1	13.5	37.5	*0.0*	*17.7*	*8.3*	*11.5*
		Gen&Tiss 2013 (2x)	85	0.0	81.2	2.4	0.0	16.5	*0.0*	*7.1*	*0.0*	*9.4*
		Gen&Tiss 2014 (2x)	85	0.0	88.2	0.0	2.4	9.4	*1.2*	*2.4*	*0.0*	*5.9*
		Gen&Tiss 2015	46	6.5	76.1	2.2	2.2	13.0	*0.0*	*6.5*	*0.0*	*6.5*
		Gen&Tiss 2016	41	14.6	68.3	2.4	0.0	14.6	*2.4*	*4.9*	*0.0*	*7.3*
		Gen&Tiss 2017	42	47.6	45.2	0.0	2.4	4.8	*0.0*	*4.8*	*0.0*	*0.0*
	**Total *EGFR***	ESP Lung EQA schemes 2013 – 2018 and Gen&Tiss Lung EQA schemes 2013-2017	**2377**	**12.0**	**52.5**	**2.6**	**2.8**	**30.2**	***5.5***	***9.6***	***4.2***	***10.9***
KRAS NM_033360.4 (Colon schemes)	c.34G>A p.(Gly12Ser)	Gen&Tiss 2013	49	0.0	93.9	6.1	0.0	0.0	*0.0*	*0.0*	*0.0*	*0.0*
	c.34G>A p.(Gly12Ser)	Gen&Tiss 2014	46	0.0	89.1	0.0	2.2	8.7	*0.0*	*2.2*	*0.0*	*6.5*
	c.35G>A p.(Gly12Asp) (3x)	Total	215	14.0	61.4	2.8	3.3	18.6	*2.8*	*6.5*	*1.4*	*7.9*
		ESP 2013	118	6.8	54.2	4.2	4.2	30.5	*5.1*	*8.5*	*2.5*	*14.4*
		Gen&Tiss 2013	49	0.0	98.0	2.0	0.0	0.0	*0.0*	*0.0*	*0.0*	*0.0*
		Gen&Tiss 2017	48	45.8	41.7	0.0	4.2	8.3	*0.0*	*8.3*	*0.0*	*0.0*
	c.35G>C p.(Gly12Ala)	Gen&Tiss 2013	46	0.0	93.5	4.3	0.0	2.2	*2.2*	*0.0*	*0.0*	*0.0*
	c.35G>T p.(Gly12Val)	ESP 2012	91	2.2	52.7	11.0	1.1	33.0	*4.4*	*17.6*	*2.2*	*8.8*
	c.38G>A p.(Gly13Asp) (5x)	Total	344	4.1	75.6	4.1	0.3	16.0	*4.9*	*5.8*	*2.6*	*2.6*
		Gen&Tiss 2013 (3x)	147	0.0	96.6	3.4	0.0	0.0	*0.0*	*0.0*	*0.0*	*0.0*
		ESP 2012	93	3.2	52.7	9.7	1.1	33.3	*4.3*	*17.2*	*2.2*	*9.7*
		ESP 2014	104	10.6	66.3	0.0	0.0	23.1	*12.5*	*3.8*	*6.7*	*0.0*
	c.176C>A p.(Ala59Glu)	ESP 2017	86	27.9	55.8	2.3	1.2	12.8	*3.5*	*2.3*	*5.8*	*1.2*
	c.183A>C p.(Gln61His) (2x)	Total	96	24.0	64.6	1.0	3.1	7.3	*0.0*	*5.2*	*0.0*	*2.1*

		Gen&Tiss 2015	48	8.3	79.2	2.1	4.2	6.3	*0.0*	*2.1*	*0.0*	*4.2*
		Gen&Tiss 2017	48	39.6	50.0	0.0	2.1	8.3	*0.0*	*8.3*	*0.0*	*0.0*
	c.183A>T p.(Gln61His) (2x)	Total	156	28.8	47.4	3.8	0.0	19.9	*3.2*	*4.5*	*0.6*	*11.5*
		ESP 2018	109	33.9	39.4	5.5	0.0	21.1	*3.7*	*3.7*	*0.9*	*12.8*
		Gen&Tiss 2016	47	17.0	66.0	0.0	0.0	17.0	*2.1*	*6.4*	*0.0*	*8.5*
	c.351A>T p.(Lys117Asn)	Gen&Tiss 2016	44	15.9	63.6	0.0	0.0	20.5	*4.5*	*6.8*	*0.0*	*9.1*
	c.437C>T p.(Ala146Val)	Gen&Tiss 2014	44	0.0	88.6	0.0	2.3	9.1	*0.0*	*2.3*	*0.0*	*6.8*
	**Total *KRAS***	ESP Colon EQA schemes 2012 – 2018 and Gen&Tiss Colon EQA schemes 2013-2017	**1217**	**12.4**	**69.5**	**3.0**	**1.3**	**13.8**	***2.5***	***5.3***	***1.2***	***4.8***
NRAS NM_002524.5 (Colon schemes)	c.34G>C p.(Gly12Arg)	Gen&Tiss 2016	41	12.2	73.2	0.0	0.0	14.6	*2.4*	*9.8*	*2.4*	*0.0*
	c.35G>A p.(Gly12Asp)	Gen&Tiss 2014	46	0.0	89.1	0.0	2.2	8.7	*0.0*	*2.2*	*0.0*	*6.5*
	c.181C>A p.(Gln61Lys) (4x)	Total	283	12.7	61.5	3.9	2.5	19.4	*3.2*	*5.3*	*1.8*	*9.2*
		ESP 2013	111	6.3	51.4	4.5	3.6	34.2	*5.4*	*8.1*	*3.6*	*17.1*
		ESP 2016	77	27.3	55.8	3.9	1.3	11.7	*3.9*	*2.6*	*1.3*	*3.9*
		Gen&Tiss 2015 (2x)	95	8.4	77.9	3.2	2.1	8.4	*0.0*	*4.2*	*0.0*	*4.2*
	c.182A>G p.(Gln61Arg)	ESP 2016	14	21.4	78.6	0.0	0.0	0.0	*0.0*	*0.0*	*0.0*	*0.0*
	c.182A>T p.(Gln61Leu)	ESP 2016	31	19.4	58.1	0.0	3.2	19.4	*6.5*	*3.2*	*3.2*	*6.5*
	c.183A>T p.(Gln61His)	Gen&Tiss 2014	44	0.0	86.4	2.3	2.3	9.1	*0.0*	*2.3*	*0.0*	*6.8*
	**Total N*RAS***	ESP Colon EQA schemes 2012 – 2018 and Gen&Tiss Colon EQA schemes 2013-2017	**459**	**11.9**	**71.3**	**1.7**	**1.8**	**13.3**	***2.3***	***4.0***	***1.3***	***5.6***

Supp. Table S2. Different categories of reported reference sequences in this study.

* Laboratories who included a correct sequence with a previous version number were not penalized. Laboratories were free to use a version number of choice, as long as it was well documented in the report or laboratory information system so it allowed an unambiguous interpretation of the variant location. They received a suggestive comment that a more recent version number is available to raise awareness. Abbreviations: EQA, External Quality Assessment; ESP, European Society of Pathology; LRG, Locus Reference Genomic; N, number; NM, protein-coding transcripts (mRNA) provided by RefSeq; NSCLC, non-small cell lung cancer; mCRC, metastatic colorectal cancer.

**EQA provider**	**ESP**				**Gen&Tiss**				**Total**	
**Indication**	**NSCLC**		**mCRC**		**NSCLC**		**mCRC**		**NSCLC**	
**Category**	**N**	**% Entries**	**N**	**% Entries**	**N**	**% Entries**	**N**	**% Entries**	**N**	**% Entries**
Correct reference sequence (LRG or NM format)	155	22%	207	32%	104	44%	105	48%	571	32%
Correct (ENST, ENSG, NG or NC format)	29	4%	25	4%	1	0%	0	0%	55	3%
Correct reference sequence present, but outdated version number*	67	10%	42	7%	32	13%	29	13%	170	9%
Reference sequence present, but no version number	37	5%	37	6%	58	24%	47	21%	179	10%
Incorrect reference sequence	17	2%	4	1%	8	3%	3	1%	32	2%
NO reference sequence	387	56%	327	51%	36	15%	35	16%	785	44%
**Total**	**692**	**100%**	**642**	**100%**	**239**	**100%**	**219**	**100%**	**1792**	**100%**

Supp. Table S3. Observed nomenclature in package inserts from commercially available techniques used by participants in the 2018 ESP EQA schemes and 2017 Gen&Tiss EQA scheme.

† Package inserts were last accessed on 01/12/2018. Abbreviations: CE-IVD, CE labeled for In vitro Diagnostic Use according to the IVD directive (Directive 98/79/EC, 1998); *EGFR*, Epidermal Growth Factor Receptor; EQA, External Quality Assessment; ESP, European Society of Pathology; HGVS, Human Genome Variation Society; *KRAS*, Kirsten ras oncogene homolog; *NRAS*, NRAS proto-oncogene GTPase; RUO, research use only. Correct nomenclature according to HGVS recommendations should be written as c.34G>C p.(Gly12Ser) for *KRAS* (NM_033360.4) and *NRAS* (NM_002524.5), and c.2369C>T p.(Thr790Met) for *EGFR* analysis (NM_005228.5).

Assay	Manufacturer	Assay status	Incorrect nomenclature format mentioned in package insert †
KRAS NM_033360.4			
KRAS Mutation Detection Kit	Amoy Dx	CE-IVD	34G>A G12S
KRAS/NRAS Detection Kit	Amoy Dx	CE	34G>A G12S
Idylla KRAS Mutation Test	Biocartis	CE-IVD	(c.34G>A) G12S
Easy KRAS mutation analysis kit	Diatech Pharmacogenetics	CE-IVD	G12S (34G>A)
Myriapod Colon Status	Diatech Pharmacogenetics	CE-IVD	Unknown
KRAS mutation analysis kit	EntroGen	Available as RUO and CE-IVD	G12S c.34G>A
KRAS/BRAF mutation analysis kit	EntroGen	Available as RUO and CE-IVD	G12S c.34G>A
RAS mutation screening panel	EntroGen	Available as RUO and CE-IVD	G12S
CLART CMA-KRAS BRAF PI3K	Genomica	CE-IVD	G12S
Therascreen KRAS Pyro Kit	Qiagen	CE-IVD	AGT G12S
Therascreen KRAS RGQ Kit	Qiagen	CE-IVD	GLY12SER (G12S) GGT>AGT
Cobas KRAS Mutation Test	Roche	CE-IVD	c.34G>A 12S and G12S
KRAS Mutation Test v2 LSR	Roche	RUO	c.34G>C p.Gly12Ser
MutectorTM KRAS Kit	Trimgen	RUO	Gly12Ser (GGT>AGT)
KRAS XL StripAssay	ViennaLab	CE-IVD	G12S
NRAS NM_002524.5			
NRAS Mutation Detection Kit	Amoy Dx	CE	34G>A G12S

KRAS/NRAS Detektion Kit AmoyDX	Amoy Dx	CE	34G>A G12S
Idylla NRAS-BRAF Mutation Test	Biocartis	CE-IVD	(c.34G>A) G12S
Idylla NRAS Mutation Test	Biocartis	CE-IVD	(c.34G>A) G12S
Idylla NRAS-BRAF-EGFRS492R assay	Biocartis	RUO	(c.34G>A) G12S
Easy NRAS mutation analysis kit	Diatech Pharmacogenetics	CE-IVD	Unknown
Myriapod Colon Status	Diatech Pharmacogenetics	CE-IVD	Unknown
NRAS Mutation Detection Kit	EntroGen	Available as RUO and CE-IVD	G12S
RAS mutation screening panel	EntroGen	Available as RUO and CE-IVD	G12S
CLART CMA-NRAS iKRAS	Genomica	CE-IVD	G12S (34 G>A)
RAS extension Pyro Kit	Qiagen	CE-IVD	34G>A G12S
Therascreen NRAS Pyro Kit	Qiagen	CE-IVD	AGT G12S
BRAF/NRAS Mutation Test (LSR)	Roche	RUO	c.34G>A G12S
KRAS/NRAS Lightmix Kit	Tib Molbiol	RUO	p.G12S c.34G>A
NRAS Mutector	Trimgen	RUO	G12S (GGT>AGT)
NRAS XL StripAssay	ViennaLab	CE-IVD	G12S
EGFR NM_005228.5			
EGFR 29 Mutations Detection Kit	Amoy Dx	CE-IVD	2369C>T T790M
QuantideX® NGS Pan Cancer Kit*	Asuragen	RUO	p.T790M
Idylla EGFR Mutation Test	Biocartis	CE-IVD	c.2369C>T T790M
Easy EGFR kit	Diatech Pharmacogenetics	CE-IVD	Unknown
Myriapod Lung Status	Diatech Pharmacogenetics	CE-IVD	Unknown
PentaPanel	Diatech Pharmacogenetics	CE-IVD	Unknown
EGFR Mutation Analysis Kit	EntroGen	CE-IVD	T790M – 2369C>T
CLART CMA EGFR	Genomica	CE-IVD	T790M (c.2369 C>T)
Therascreen EGFR Pyro Kit	Qiagen	CE-IVD	ATG T790M

Therascreen EGFR RGQ PCR Kit	Qiagen	CE-IVD	T790M 2369C>T
Cobas EGFR Mutation detection Test v1	Roche	CE-IVD	2369C>T T790M
Cobas EGFR Mutation detection Test v2	Roche	CE-IVD	2369C>T T790M
EGFR XL StripAssay	ViennaLab	CE-IVD	T790M
